# Presentation to Miss Ray

**Published:** 1918-12-14

**Authors:** M. E. Ray


### Presentation to Miss Ray.

*To the Editor of* The Hospital.

Sir,—May I, through your columns, express my very warm thanks to the many friends who have so kindly given me such a handsome testimonial? I am afraid it will not be possible for me, at present, to thank everyone personally, but I hope that I may, in the future, have many opportunities of meeting my friends in the nursing world, as although I have relinquished the responsible post I held at "King's," my interest in, and sympathy with, everything connected with nursing continues unabated, and I hope that in a smaller way I may still be of some use to the profession I love. It will be a great source of joy to me in my own home to feel that I am surrounded with mementoes of those with whom I have lived and worked for so many years.—I am, dear Sir, yours very truly,

M. E. Ray.

December 6, 1918.

